# Femoral–Femoral Artery Artificial Vascular Bypass: A Promising Therapy for Infectious Arteritis After Kidney Transplantation

**DOI:** 10.1155/joot/9632239

**Published:** 2026-06-03

**Authors:** Jiangwei Zhang, Minyue Zhang, Enbo Li, Xiaoming Ding

**Affiliations:** ^1^ Department of Kidney Transplantation, The First Affiliated Hospital of Xi’an Jiaotong University, Xi’an, 710061, China, xjtu.edu.cn

**Keywords:** carbapenem-resistant *Enterobacteriaceae* (CRE), carbapenem-resistant *Pseudomonas aeruginosa* (CRPA), femoral-to-femoral artery artificial vascular bypass (Fem-Fem bypass), infectious arteritis, kidney transplantation, mucormycosis

## Abstract

**Objective:**

Infectious arteritis involving the iliac arteries is a rare but severe complication after kidney transplantation, frequently leading to graft loss and mortality. Direct repair within infected fields is often unsuccessful. We report outcomes of femoral–femoral artery artificial vascular bypass (Fem–Fem bypass) as a strategy to exclude infected arterial segments and preserve limb perfusion in five such cases.

**Method:**

Retrospective analysis of four kidney transplant recipients who developed infectious arteritis (external iliac artery) with rupture or aneurysm between January 2015 and January 2023. All underwent resection of the infected arterial segment with proximal/distal ligation and extra‐anatomic Fem–Fem bypass using a synthetic graft.

**Results:**

Among the four patients, two were infected with carbapenem‐resistant *Pseudomonas aeruginosa* (CRPA), one with carbapenem‐resistant Enterobacteriaceae (CRE), and one with mucormycosis. All underwent Fem‐Fem bypass following resection of the infected arterial segment. One patient with CRPA infection died 5 months postoperatively due to septicemia and multiorgan failure. The remaining three patients survived with functional grafts and patent bypasses at follow‐up, without recurrence of infection or vascular complications.

**Conclusions:**

Fem–Fem bypass effectively controls hemorrhage or aneurysm expansion and maintains limb perfusion by excluding the infected iliac arterial segment in kidney transplant recipients with infectious arteritis. It represents a viable salvage option when endovascular repair fails or is contraindicated. Long‐term success depends on effective infection control, appropriate antimicrobial therapy, and patient‐specific factors. Mortality remains significant in cases with systemic sepsis or highly resistant pathogens.

## 1. Introduction

Kidney transplantation stands as the gold standard treatment for end‐stage kidney disease (ESKD), offering superior survival and quality of life compared to long‐term dialysis [1, 2]. Contemporary immunosuppressive regimens and refined surgical techniques have led to remarkable outcomes, with 1‐year graft survival rates now exceeding 95% and 10‐year survival approaching 60%–70% in many centers [3–5]. Vascular complications occur in 1.35% of cases [6], among which infectious arteritis is particularly catastrophic, carrying graft loss rates of 75%–100% and mortality rates of 25%–50% [7–9]. This devastating outcome is often driven by arterial rupture and uncontrolled sepsis in the setting of an immunocompromised host.

The most common pathogens implicated include bacteria such as *Staphylococcus aureus, Pseudomonas aeruginosa,* and Enterobacteriaceae, as well as fungi including *Candida, Aspergillus,* and Mucorales. Conventional treatments (such as graft nephrectomy, arterial ligation, and debridement) frequently result in limb ischemia or loss, while endovascular stenting carries a high failure rate in the setting of active infection.

This report details four instructive cases managed with an aggressive limb‐ and potentially graft‐salvaging strategy: resection and ligation of the infected iliac arterial segment combined with an extra‐anatomic femoral–femoral artery artificial vascular bypass (Fem–Fem bypass). The rationale for this approach lies in its ability to exclude the infected arterial segment, restore limb perfusion via an extra‐anatomic route, and avoid direct reconstruction within the contaminated field. We significantly expand the original discussion to delve into the technical intricacies of the bypass procedure, the formidable challenge of carbapenem‐resistant *Pseudomonas aeruginosa* (CRPA), carbapenem‐resistant Enterobacteriaceae (CRE), and mucormycosis management in immunocompromised hosts, the critical factors differentiating success from failure, and the broader context of managing this devastating complication based on current literature and evolving best practices. We aim to provide a comprehensive resource for transplant surgeons and infectious disease specialists confronting this rare but high‐stakes scenario.

## 2. Patients and Methods

### 2.1. Patients

This study is a single‐center, retrospective case series. Between 2015 and 2023, 3218 kidney transplants were performed at our institution; four recipients developed infectious arteritis involving the external iliac artery and underwent Fem–Fem bypass.

Inclusion criteria are as follows: (1) Confirmed or highly suspected infective arteritis involving the external iliac artery (may extend to the anastomosis or the main kidney artery of the graft); (2) evidence by imaging or intraoperative findings of arterial rupture, pseudoaneurysm, or true aneurysm with risk of bleeding or embolism; (3) safe in situ arterial reconstruction is not feasible due to any of the following: Full‐thickness arterial wall involvement by infection, with fragile, edematous, or necrotic tissue; (4) infection focus adjacent to the transplanted kidney or persistent infection after graft nephrectomy; (5) the contralateral iliofemoral system is patent (no ≥ 50% stenosis or occlusion) and can serve as a source of inflow; (6) the ipsilateral common femoral artery and distal runoff (profunda femoris and superficial femoral arteries) are patent and able to accept retrograde blood perfusion; and (7) endovascular stenting has failed or is contraindicated (e.g., active infection and mycotic aneurysm). Exclusion criteria are as follows: (1) uncontrolled systemic sepsis or multiple organ failure; (2) severe stenosis or occlusion of the contralateral iliac or femoral artery, precluding adequate inflow; (3) no suitable distal runoff on the affected side (e.g., severe occlusive disease); and (4) extremely poor life expectancy such that the patient would not benefit from surgery. Four patients underwent kidney transplantation from deceased donors (DDs) and received a standard immunosuppressive regimen consisting of tacrolimus, mycophenolate mofetil, and prednisone [10]. We performed the transplant through a classic Gibson incision and located the kidney graft in the right iliac fossa. Proactive anti‐infection therapy was administered both pre‐ and postoperatively. In cases where pathogen cultures returned positive, treatment adjustments were made according to drug sensitivity testing. We retrospectively analyzed their clinical data, management course, and follow‐up outcomes (Table 1).

**TABLE 1:** Demographic and clinical characteristics of four patients.

Patient	Transplant type	Age/Sex	Time of onset	Artery anastomosis	Perioperative immunosuppression protocol	Oral immunosuppressive regimen	Clinical symptom	Postoperative complication	Possible risk factor
1	DD	39/M	10 days	External iliac	ATG‐F	Tac + MMF	Fever, massive bleeding	Acute rejection	DDI
2	DD	33/M 0.07	3 months	Internal iliac	rATG	CsA + MMF	Fever, pain	COVID‐19	Secondary transplant
3	DD	59/F	14 days	External iliac	Basiliximab	Tac + MMF	Perirenal hematoma	—	Innutrition, diabetes mellitus
4	DD	48/M	7 days	External iliac	ATG‐F	Tac + MMF	Massive bleeding	DGF	Unclear

*Note:* Tac, tacrolimus; CsA, Cyclosporin A; MMF, mycophenolate mofetil; ATG‐F, anti‐human T lymphocyte rabbit immunoglobulin; rATG, rabbit anti‐human thymocyte globulin; COVID‐19, coronavirus disease 2019.

Abbreviations: DD, deceased donor; DDI, donor‐derived infection; DGF, delayed graft function.

### 2.2. Surgical Technique

The core strategy involved the following: (1) Resection of the infected external iliac artery segment and thorough debridement; (2) ligation of the proximal and distal arterial stumps; and (3) creation of an extra‐anatomic subcutaneous Fem–Fem bypass using a synthetic graft (e.g., PTFE or Dacron) to restore limb perfusion, avoiding the infected field.

#### 2.2.1. Resection of the Infected External Iliac Artery Segment and Thorough Debridement

After graft nephrectomy, the infected segment of the external iliac artery is thoroughly resected and debrided. The infected arterial segment is carefully dissected from surrounding adhesive tissues, avoiding injury to adjacent structures such as the ureter, iliac vein, and nerves. The full thickness of the infected arterial wall is completely excised until both resection margins show normal, healthy arterial wall characteristics (smooth intima, free of edema and necrosis). Subsequently, the residual infected bed is aggressively debrided to remove all necrotic tissue, purulent exudate, and foreign material. The surgical field is repeatedly irrigated with copious amounts of dilute povidone–iodine solution and normal saline to reduce local bacterial load. Surgical instruments and gloves are replaced aseptically during the procedure to strictly isolate the infected area from the subsequent reconstruction site.

#### 2.2.2. Ligation of the Proximal and Distal Arterial Stumps

After resection of the infected segment, nonabsorbable sutures are used to doubly ligate or suture‐ligate the proximal and distal arterial stumps, ensuring complete closure of the lumen to prevent postoperative bleeding or extension of residual infection along the artery. Prior to ligation, it is confirmed that the intima of the stumps is intact and there is no active bleeding from the surrounding tissues. For the proximal stump, a temporary vascular clamp may be applied; the clamp is removed after ligation is completed and confirmed to be secure. The distal stump is managed similarly. After ligation, the peristump tissues are re‐examined, and if necessary, adjacent healthy soft tissue or an omental flap is used to cover and reinforce the stumps, isolating them from the infected environment. This step aims to completely occlude blood flow through the infected vessel and create a clean, infection‐free anatomical field for subsequent bypass surgery.

#### 2.2.3. Fem–Fem Bypass

A 5‐cm oblique incision was made along the left groin crease. The left common femoral artery, profunda femoris artery, and superficial femoral artery were exposed through layered dissection. The proximal common femoral artery and distal profunda femoris artery/superficial femoral artery were clamped. A 1‐cm longitudinal arteriotomy was made in the common femoral artery. An end‐to‐side anastomosis was performed between the common femoral artery and the artificial vascular graft. Using the same approach, a 5‐cm oblique incision was made along the right groin crease to expose the right common femoral artery, profunda femoris artery, and superficial femoral artery. The distal end of the graft was tunneled subcutaneously to the right side. After trimming the excess graft, the right profunda femoris artery, superficial femoral artery, and common femoral artery were clamped. An end‐to‐side anastomosis was performed between the graft and the right common femoral artery. Upon clamp release, the graft filled well with pulsatile flow, and both anastomoses were hemostatic (Figure 1A). The wounds were closed with local compression applied. Strict aseptic technique was maintained throughout, with instrument and glove changes before handling the new graft.

**FIGURE 1:**
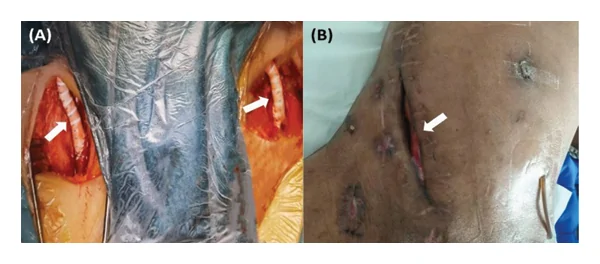
The diagnosis and treatment process of Patient 1. (A) Schematic of Fem–Fem bypass performed for emergent hemorrhage control. (B) Postoperative course complicated by uncontrolled CRPA infection and delayed wound healing, ultimately leading to fatal sepsis.

## 3. Results

### 3.1. Patient 1

Patient 1 presented with acute arterial rupture at the iliac anastomosis 13 days posttransplant. Emergency surgery (graft nephrectomy, infected external iliac artery resection, and Fem–Fem bypass) successfully controlled hemorrhage. CRPA was isolated from cultures (Table S1). Despite targeted antimicrobial therapy (polymyxin/tigecycline), the patient developed persistent sepsis and died 5 months later from multiorgan failure (Figure 1B).

### 3.2. Patient 2

Patient 2 presented 3 months posttransplant with fever and a right external iliac artery aneurysm, with concurrent CRPA bacteremia. After failed endovascular stenting, open surgical resection and Fem–Fem bypass were performed. At 2‐year follow‐up, the patient remained infection‐free with a functional graft and patent bypass (Figure 2).

**FIGURE 2:**
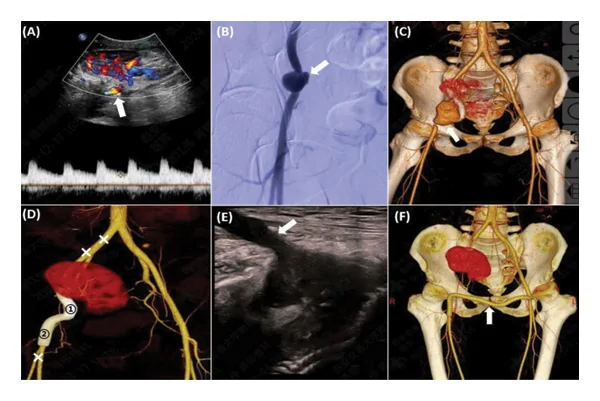
The diagnosis and treatment process of Patient 2. (A) Ultrasound showing external iliac artery aneurysm. (B) Angiography confirming aneurysm. (C) New aneurysm distal to the first stent. (D) Placement of the second stent graft. (E) Abscess/sinus tract formation around stented aneurysm. (F) Postoperative CTA showing occluded native external iliac artery, patent Fem–Fem bypass graft, and viable kidney graft.

### 3.3. Patient 3

A 59‐year‐old female developed CRE‐infected arteritis with rupture 1 month posttransplant. She was treated with Fem–Fem bypass and combination antibiotics (including ceftazidime–avibactam). She survived with a patent bypass and stable graft function at 6‐year follow‐up.

### 3.4. Patient 4

A 48‐year‐old male presented with mucormycosis‐related arteritis and aneurysm 4 months posttransplant. He underwent surgical resection, Fem–Fem bypass, and long‐term liposomal Amphotericin B therapy. He survived with no signs of recurrence at 5‐year follow‐up.

## 4. Discussion

Organ transplantation is currently regarded as the most effective treatment for end‐stage organ failure, with kidney transplantation being the earliest, most frequently performed, and most successful type [11]. Common vascular complications after kidney transplantation include stenosis, dissection (involving iliac or transplant kidney arteries), infectious arteritis, pseudoaneurysm, rupture, and thrombosis [12]. Bessede et al. confirmed that vascular complications correlate with reduced graft survival, accounting for 1.66% of graft losses and 0.16% of recipient deaths [13]. Therefore, early detection, diagnosis, and treatment of posttransplant vascular complications are crucial.

Infectious arteritis is a condition in which pathogens directly infect the kidney artery (typically at the anastomotic site) or its major branches, leading to arterial wall inflammation, destruction, and the formation or rupture of an aneurysm (mycotic aneurysm). The core mechanism involves the colonization and destruction of the arterial wall by pathogens. Infectious arteritis after kidney transplantation is an extremely devastating complication, often resulting in graft loss or patient death. Its occurrence is closely related to posttransplant immunosuppression, surgical procedures, and increased infection risk [14, 15]. Vascular rupture due to infectious arteritis typically occurs at the anastomosis. After reviewing all the cases, we observed certain variations in the locations of arteritis. In Patient 1 and Patient 3, the lesions were situated at or directly involved the vascular anastomotic site, while in Patient 4, the lesion was clearly located on the main kidney artery trunk of the graft distal (below) to the anastomosis. This difference in location may suggest distinct pathophysiological mechanisms or disease progression pathways, warranting further investigation. Although Patient 2 did not exhibit arteritis directly related to the transplanted kidney, the issue of peritransplant vascular infection was successfully resolved via Fem–Fem bypass, demonstrating the broad applicability of this surgical technique. Infectious arteritis clinical presentation includes sudden pain and swelling in the graft area, progressively enlarging hematoma, localized tenderness (which may radiate to the lower back or abdomen), oliguria, and progressive decreases in hemoglobin and blood pressure. Severe cases may present with shock, and drainage is bloody. We now reference studies that report poor outcomes with direct arterial repair or endovascular stenting in infected fields and highlight that extra‐anatomic bypass, though well‐established for aortoiliac infections, has been rarely described in the context of kidney transplant–associated infectious arteritis. Our series is one of the few to specifically apply femoral–femoral bypass in this setting, with long‐term follow‐up in survivors, offering a unique perspective on limb‐ and graft‐salvage in this high‐risk population.

In all four cases, the infection involved the external iliac artery, making direct reconstruction unsafe. Alternative approaches considered included the following: Axillofemoral bypass, which was deemed less favorable due to longer graft length, higher occlusion rates, and the need for additional incisions. Autologous vein reconstruction, which was avoided due to the risk of graft infection and the lack of suitable autologous conduit in these immunocompromised patients. Endovascular stenting, which was attempted in one patient (Patient 2) and failed, reinforces the need for open surgical exclusion. For refractory infectious arteritis involving the external iliac artery, we employed a strategy of infection site exclusion and vascular reconstruction: surgical removal of the infectious source, thorough debridement, ligation of the proximal and distal ends of the infected artery, and Fem–Fem bypass to restore blood supply to the affected limb. The femoral–femoral bypass was selected because it offers a short, straightforward extra‐anatomic route. The core advantage of Fem–Fem bypass is its ability to circumvent the infected area, which is paramount [16, 17]. Traditional approaches involving direct repair on infected, fragile, and inflamed arteries carry an extremely high risk of failure, as evidenced by the failed stent graft procedures in Patient 2 [12]. Fem–Fem bypass is performed in a noninfected tissue plane (subcutaneous tunnel), completely isolating the infected zone and establishing a new lower limb blood flow pathway in a “clean” plane. In this study, this technique successfully controlled life‐threatening bleeding and progressive aneurysm enlargement, with no recurrence of local hemorrhage.

Fem–Fem bypass is typically used for chronic lower limb ischemic disease, rarely used for posttransplantation infectious arteritis. Indications include unilateral external iliac artery occlusion with good distal femoral artery runoff, a contralateral external iliac artery without significant stenosis, and patients in poor general condition or with failed endovascular intervention [7]. The procedure is relatively simple, minimally invasive, and avoids the abdominal cavity (thus eliminating abdominal complications), but carries risks of graft stenosis and thrombosis. Intraoperative attention should be paid to the position of the healthy femoral artery; anastomosis should be slightly higher than that of the diseased side, and the anastomosis should be made on the inner side of the femoral artery to avoid the angulation of the transplanted vessel. Excessive angulation may lead to graft thrombosis and occlusion. Literature reports 3‐year patency rates ranging from approximately 60% to 80% [7, 18–20]. We monitor the artificial blood vessel and lower limb blood flow every 3 months through color Doppler ultrasound and routinely administer aspirin or clopidogrel for anticoagulation after surgery.

What explains the differing outcomes in the four patients? Patient 1 experienced anastomotic rupture merely 10 days posttransplant, likely during the peak period of immunosuppression. Having undergone significant surgical trauma, the patient had extremely low resistance. The pathogen causing Patient 1’s infection originated from a donor‐derived infection’s CRPA strain, which exhibits high virulence, and anti‐infective therapy was suboptimal, allowing rapid infection progression. While surgery addressed the immediate life‐threatening local problem, severe sepsis and subsequent MODS were likely the primary causes of death [9, 21]. This case represented an emergency life‐saving procedure; the surgery itself was successful, but it could not reverse the fatal consequences of systemic infection, highlighting the systemic threat of such severe infections and the patient’s overall fragility. Among the three surviving patients, none underwent retransplantation during follow‐up (2–6 years). In all three, the femoral–femoral bypass remained patent and was not taken down; there was no reconstruction of the native iliac artery, as the bypass provided adequate limb perfusion without complications.

Our clinical practice offers several important insights: (1) Early recognition and potent anti‐infective therapy are fundamental. The success of any vascular reconstruction surgery hinges on effective infection control. (2) Infection site exclusion is a gold standard for managing infectious vasculitis. When medications and limited debridement fail to control the infection, complete exclusion of the infected vascular segment is necessary and effective. (3) Fem–Fem bypass is a reliable option, particularly when the external iliac artery requires exclusion due to infection. This technique provides an extra‐anatomic bypass, is relatively mature, and yields dependable results. (4) Comprehensive patient assessment is critical. Surgical decision‐making must balance the urgency of the local problem, infection severity, the patient’s systemic condition, and tolerance. Patient 1’s outcome reminds us that even successful local surgery cannot overcome the threat of severe systemic infection and MODS. (5) Individualized treatment is essential, requiring a tailored approach based on infection characteristics, patient status, bleeding risk, and limb ischemia risk.

## 5. Conclusion

Fem–Fem bypass is an effective therapeutic strategy for managing infectious arteritis after kidney transplantation, particularly in cases involving resistant pathogens or fungal infections. Our expanded series demonstrates that with timely surgery, appropriate antimicrobial therapy, and careful patient selection, favorable outcomes can be achieved. Future efforts should focus on early diagnosis, optimized immunosuppression, and novel antimicrobial strategies to further improve survival in these high‐risk patients. This study provides valuable clinical experience for managing this rare but fatal complication. Currently, the small sample size and single‐center design limit generalizability, with few global reports. Long‐term bypass patency (> 5 yr) remains unevaluated.

## Author Contributions

Jiangwei Zhang and Xiaoming Ding: conceptualization of the research design, execution of data collection, data analysis and visualization, and principal writer. Minyue Zhang and Enbo Li: conceptualization of the research design, writing, and editing.

## Funding

No funding was received for this study.

## Ethics Statement

This study was conducted in accordance with the principles of the Declaration of Helsinki and approved by the Institutional Review Board of The First Affiliated Hospital of Xi’an Jiaotong University (Approval No. XJTU1AF2023LSK‐2022–178). All kidneys used in this study were obtained from deceased donors through the legally regulated organ donation system of China, with written informed consent obtained from the donor families or legally authorized representatives. No organs from executed prisoners or other vulnerable populations were used. Written informed consent for participation and publication of clinical data was obtained from all kidney transplant recipients. No donor‐specific identifiable data were collected or analyzed in this study.

## Consent

Please see the Ethics Statement.

## Conflicts of Interest

The authors declare no conflicts of interest.

## Supporting Information

Additional supporting information can be found online in the Supporting Information section.

## Supporting information


**Supporting Information** Table S1 summarizes the pathogen culture and antimicrobial susceptibility testing (AST) results for the isolate from Patient 1. The minimum inhibitory concentrations (MICs) of 15 antibiotics were determined, and the results were interpreted as susceptible (S), intermediate (I), or resistant (*R*) according to CLSI or corresponding susceptibility breakpoints. The results showed that the isolate was resistant to multiple clinically commonly used antibiotics (e.g., cefoperazone/sulbactam, meropenem, ciprofloxacin, and amikacin) and was only susceptible to colistin (MIC ≤ 0.5 μg/mL), indicating an extensively drug‐resistant strain. These AST findings provided an important basis for selecting subsequent anti‐infective treatment strategies (see Supporting Table S1).

## Data Availability

The data that support the findings of this study are available from the corresponding author upon reasonable request.
