# Supplementary material for: Femoral–Femoral Artery Artificial Vascular Bypass: A Promising Therapy for Infectious Arteritis After Kidney Transplantation
Source: J Transplant. 2026 Jun 3;2026:9632239. doi: 10.1155/joot/9632239 (PMC13747090; doi:10.1155/joot/9632239)
Supplement: Supplementary file 1 [file JOOT-2026-9632239-s001.docx]

**Table S1. Results of pathogen culture and antimicrobial susceptibility test of patient 1**

| Antibiotic | MIC（ug/ml） | Range of the Breakpoint | Sensibility | Antibiotic | MIC（ug/ml） | Range of the Breakpoint | Sensibility |
| --- | --- | --- | --- | --- | --- | --- | --- |
| Cefoperazone/Sulbactam | >=64.0 |  | (R) | Piperacillin/tazobactam | >=128 | 16-128 | (R) |
| Ceftazidime | >=64 | 8-32 | (R) | Cefepime | >=32 | 2-16 | (R) |
| Aztreonam | 21 | 15-22 | (I) | Meropenem | >=16 | 2-8 | (R) |
| Imipenem | >=16 | 2-8 | (R) | Ciprofloxacin | >=16 | 0.25-1 | (R) |
| Amikacin | >=64.0 | 16-64 | (R) | SMZ-TMP | >=320 | 2-4 | (R) |
| Levofloxacin | >=8.0 | 1-4 | (R) | Tobramycin | >=16 | 4-16 | (R) |
| Colistin | <=0.5 | <=2 | (S) | Minocycline | >=16 | 0.24 | (R) |

MIC, minimum inhibitory concentration; R, resistance; S, susceptible; I, intermediate
